# Seasonal overturn and stratification changes drive deep-water warming in one of Earth’s largest lakes

**DOI:** 10.1038/s41467-021-21971-1

**Published:** 2021-03-16

**Authors:** Eric J. Anderson, Craig A. Stow, Andrew D. Gronewold, Lacey A. Mason, Michael J. McCormick, Song S. Qian, Steven A. Ruberg, Kyle Beadle, Stephen A. Constant, Nathan Hawley

**Affiliations:** 1 Great Lakes Environmental Research Laboratory, Office of Oceanic and Atmospheric Research, National Oceanic and Atmospheric Administration, Ann Arbor, MI USA; 2 School for Environment and Sustainability, University of Michigan, Ann Arbor, MI USA; 3 Department of Environmental Sciences, The University of Toledo, Toledo, OH USA

**Keywords:** Environmental sciences, Limnology

## Abstract

Most of Earth’s fresh surface water is consolidated in just a few of its largest lakes, and because of their unique response to environmental conditions, lakes have been identified as climate change sentinels. While the response of lake surface water temperatures to climate change is well documented from satellite and summer in situ measurements, our understanding of how water temperatures in large lakes are responding at depth is limited, as few large lakes have detailed long-term subsurface observations. We present an analysis of three decades of high frequency (3-hourly and hourly) subsurface water temperature data from Lake Michigan. This unique data set reveals that deep water temperatures are rising in the winter and provides precise measurements of the timing of fall overturn, the point of minimum temperature, and the duration of the winter cooling period. Relationships from the data show a shortened winter season results in higher subsurface temperatures and earlier onset of summer stratification. Shifts in the thermal regimes of large lakes will have profound impacts on the ecosystems of the world’s surface freshwater.

## Abstract (web summary)

This study presents hourly data from a thermistor string in Lake Michigan, inspecting its response at depth to surface warming. Based on the data, the study suggests bottom lake temperatures respond to changes in turnover and re-stratification, with the ultimate possibility of the lake shifting from dimictic to monomictic.

## Introduction

Eighty-four percent of Earth’s non-frozen, surface freshwater is found in the 10 largest lakes^1^. Because of the sensitivity to changing conditions, along with the ability to integrate climate conditions across the watershed and produce measurable signals of climate-impacted parameters, lakes have been identified as ideal climate change sentinels^2–4^. As air temperatures trend upward, global lake surface water temperatures (LSWT) have warmed by an average of 0.21 °C/decade^5–9^. Several lakes appear to be warming faster than ocean temperatures (0.12 °C/decade) and regional air temperatures (0.25 °C/decade), including the world’s largest lakes^6,7,10–12^. While an abundance of surface measurements reveal surface warming in response to climate change^13^, subsurface observations are relatively sparse and may tell a story that is more indicative of long-term climate change impacts. Subsurface waters in deep lakes can provide an important signal because they integrate conditions across years, providing a climate memory^14^, and can help identify the potential for ecological and thermal shifts, e.g. from dimictic to monomictic mixing conditions^2,3,15^.

Subsurface conditions in the largest lakes, hereby referred to as large lakes, are a missing piece in the global climate change narrative. Most of our understanding of large lake warming trends comes from observations of summer LSWT due to data availability restrictions in satellite measurements, buoy deployment schedules, and seasonal ice conditions^6,7,16–19^. There has been an improved understanding of year-round or seasonal surface trends from satellite remote sensing in recent years that demonstrates the physical drivers of surface warming and the spatial and temporal variation of LSWT warming and overturn in the Laurentian Great Lakes^20–22^. However, these trends in surface temperatures are not easily translated into the more complex deep subsurface conditions, where stratification, thermocline depth, and density gradient influence subsurface mixing. Therefore, direct observations of subsurface thermal structure are imperative to the interpretation of climate impacts on the majority of Earth’s freshwater^2^.

Measurements of hypolimnetic water temperatures suitable for long-term trend analysis are limited to a few studies in deep lakes^10,12,14,23–28^, although, projected connections between climate change induced thermal structure changes and mixing regime shift have been demonstrated through numerical modeling^15,29^. Therefore, our understanding of how our largest lakes are responding to climate change at depth has been formed either by translating what has been observed at the lake surface or by subsurface observations that are limited in vertical resolution (depth), temporal frequency (e.g. weekly, monthly, etc.), or of insufficient duration for long-term analysis.

Here we show how seasonal changes in the timing of overturn and stratification link surface warming trends to deep water temperatures in a large dimictic lake using three decades of nearly continuous subsurface water temperature observations in Lake Michigan. High-frequency measurements reveal otherwise undetectable deep water dynamics during a noted period of warming in the world’s lakes. With this unique data set, we address the following questions: How do the deep waters of Earth’s largest lakes respond to climate trends? As surface temperatures rise and summer periods are extended, what winter subsurface characteristics are altered? What can high-resolution and high-frequency observations tell us that is obscured by other long-term freshwater data sets? We find the long-term data set not only confirms that deep waters are warming, but also shows how surface trends manifest into a cascade of thermal changes during the winter period. Subdaily observations detail the relationships between fall overturn, winter cooling period duration, and subsurface temperatures. Through this lens, we can see the potential for a thermal regime shift^15^ and extend the current understanding of summer LSWT trends^13^ to observed changes in other seasons and to the deep waters of Lake Michigan.

## Results and discussion

### Subsurface response to warming

**Fig. 1:**
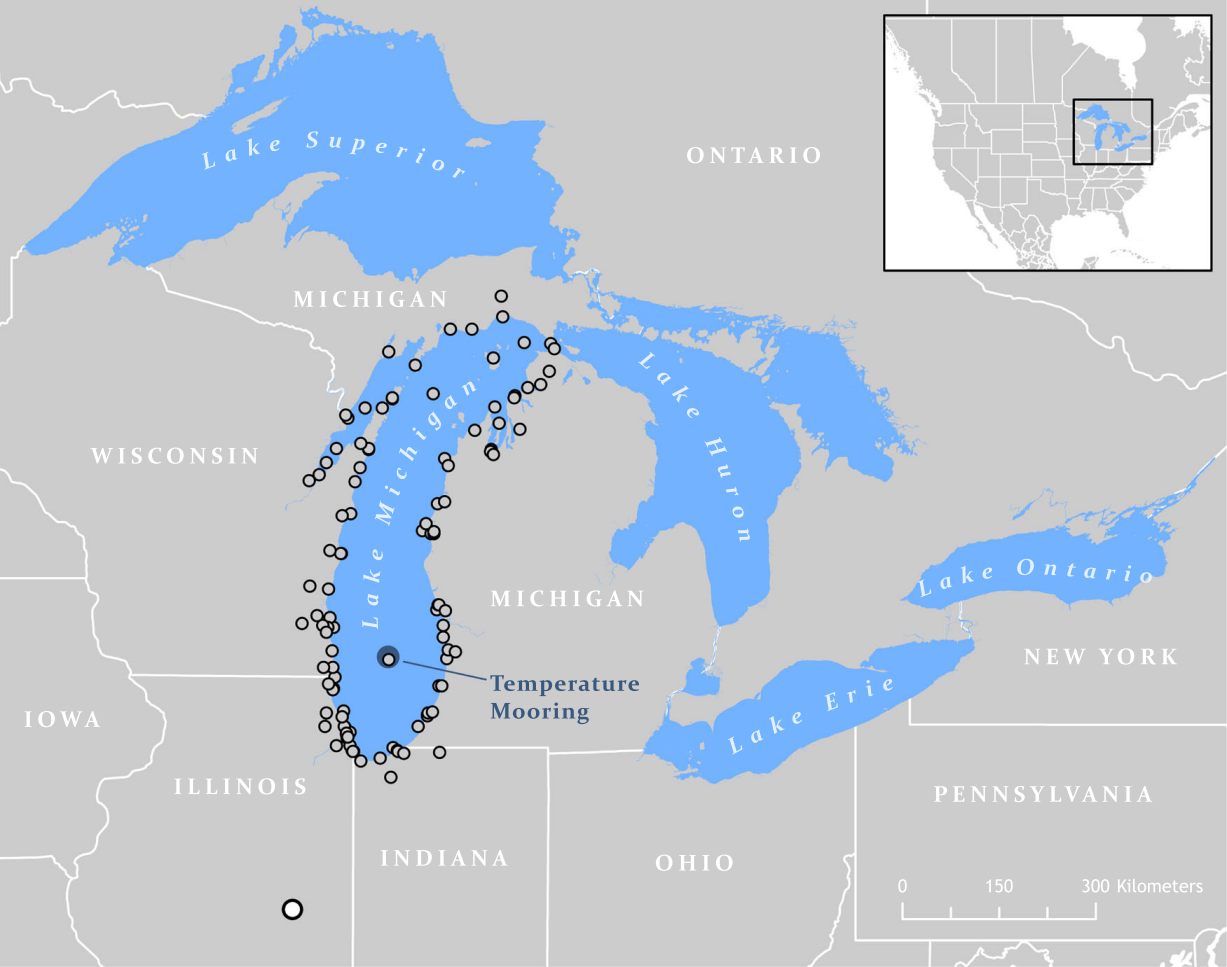
Location of the long-term temperature mooring in Lake Michigan. Three decades of high-frequency subsurface temperature measurements (1990—present) are recorded by a thermistor string at the southern Lake Michigan mooring site. Observations of atmospheric conditions are recorded at several coastal meteorological stations (gray circles) archived by NOAA. Long-term downward shortwave radiation is measured by the NOAA Surface Radiation (SURFRAD) Network (white circle) in Bondville, IL, USA.

To analyze water temperature trends in Lake Michigan (the 4th largest lake on Earth), we use 30 years of nearly continuous hourly and 3-hourly measurements from a high vertical-resolution thermistor string in 150 m of water (Fig. 1, and Supplementary Information). To place subsurface measurements in context with surface warming trends, these data were supplemented with satellite-derived surface temperature from the same location using the Great Lakes Surface Environmental Analysis (GLSEA)^30^. Previous reports of warming rates in Lake Michigan were based on summer (July–August–September) observations at the surface, via satellite and in situ buoy measurements, and estimate lake-averaged trends between 0.20 and 0.70 °C/decade^6,17^. As context for this analysis, we first updated the lake-averaged trend using satellite-derived temperatures for 1995–2019^30^ and three statistical approaches. Linear estimates of warming trends yielded year-round lake-averaged surface warming rates of 0.34 and 0.31 °C/decade, using simple linear regression and the Theil–Sen estimator, respectively. A seasonal trend decomposition (STL) method estimated an overall long-term warming trend slightly higher than the linear models, with a trend of 0.40 °C/decade (Table 1).

**Table 1:** Long-term water temperature trends at the lake surface and subsurface transects.

	Linear regression	Theil–Sen	STL
Lake-average year-round surface trend	0.34 ± 0.21	0.31 ± 0.18	0.40 ± 0.05
Mooring year-round surface trend	0.40 ± 0.26	0.41 ± 0.20	0.49 ± 0.07
Mooring year-round 30 m trend	0.07 ± 0.18*	0.11 ± 0.13*	−0.02 ± 0.05*
Mooring year-round 60 m trend	0.11 ± 0.09	0.11 ± 0.07	0.09 ± 0.03
Mooring year-round 75 m trend	0.12 ± 0.07	0.11 ± 0.06	0.11 ± 0.02
Mooring year-round 100 m trend	0.11 ± 0.06	0.08 ± 0.04	0.11 ± 0.02
Mooring year-round 110 m trend	0.05 ± 0.06*	0.04 ± 0.04*	0.06 ± 0.01
Mooring year-round 140 m trend	0.00 ± 0.08*	−0.04 ± 0.05*	0.00 ± 0.01*

Estimates are given for three statistical approaches, simple linear regression, a Theil–Sen line, and a linear fit to the STL long-term component with 95% confidence intervals in °C/decade. Confidence intervals that include zero are indicated with “*”.

**Fig. 2:**
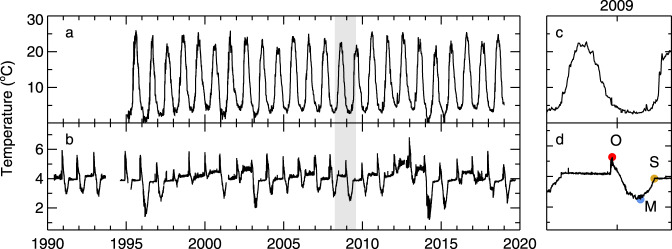
Surface and subsurface water temperature observations in Lake Michigan. **a** Daily lake surface temperature from the GLSEA at the thermistor location. **b** Water temperature time-series at the 110 m depth transect. **c**, **d** A zoomed surface and subsurface temperature record for 2008–2009, equivalent to the period highlighted in gray in **a** and **b**, indicating the 110 m overturn date, “O”, minimum temperature/date, “M”, and stratification temperature/date, “S”.

**Fig. 3:**
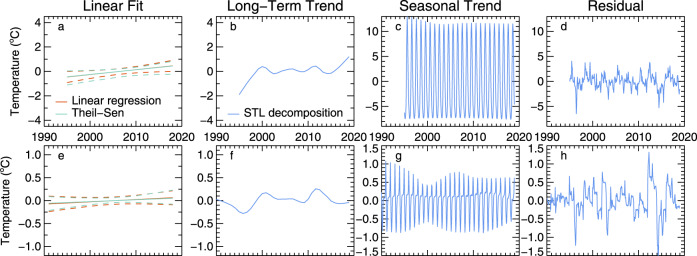
Long-term and decomposed trends in water temperature. **a** Lake surface water temperature trends based on annual GLSEA data at the thermistor location computed using two methods: simple linear regression (0.40 ± 0.26 °C/decade trend and 95% confidence interval) and a Theil–Sen line (0.41 ± 0.20 °C/decade trend and 95% confidence interval). **b** The decomposed long-term trend component of the seasonal trend decomposition (STL) method for the GLSEA lake surface temperature, a non-linear counterpart to the linear regression methods (trend from linear fit is given in Table 1). **c** Seasonal trend component from the STL for the GLSEA lake surface temperature. **d** Residual component from the STL for the GLSEA lake surface temperature. **e** Subsurface water temperature trends at 110 m depth based on a linear regression (0.05 ± 0.06 °C/decade trend and 95% confidence interval) and Theil–Sen line (0.04 ± 0.04 °C/decade trend and 95% confidence interval). **f** Long-term trend computed from the STL for the 110 m depth (trend from linear fit is given in Table 1). **g** Seasonal trend component from the STL for the 110 m depth. **h** Residual component from the STL for the 110 m depth.

**Fig. 4:**
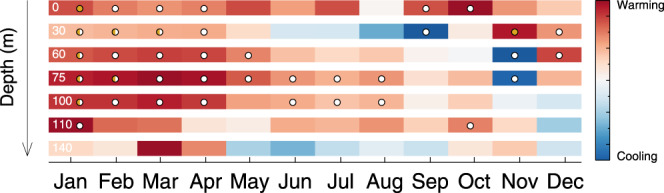
Monthly long-term water temperature trends in Lake Michigan. Monthly warming or cooling trends from the 1990–2019 record as a function of depth in the water column. For a given depth, monthly trends are normalized by the maximum of the trend magnitudes at that depth. Therefore, the darkest red and darkest blue colors represent the greatest warming and cooling rates, respectively, for a given depth. Dots indicate confidence intervals on the trend that do not include zero for 1.96 (gold circle), 1.645 (half-gold circle), and 1 (white circle) standard deviations.

**Fig. 5:**
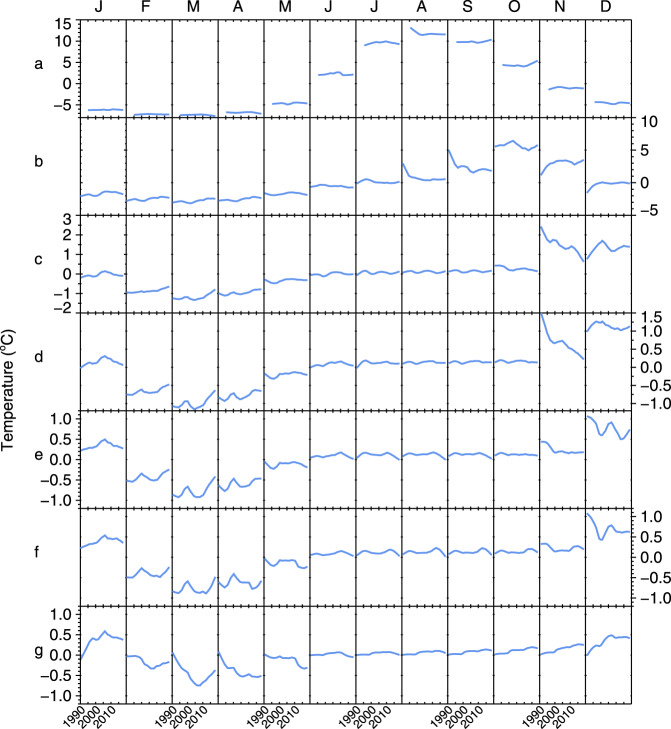
Seasonal component of decomposed water temperature. Monthly water temperature trends from the STL seasonal component as a function of depth for **a** surface, **b** 30 m, **c** 60 m, **d** 75 m, **e** 100 m, **f** 110 m, and **g** 140 m. For each month (abbreviated by first initial), the *x*-axis represents the range 1990–2019.

Applying the same approach at the mooring location in southern Lake Michigan (Fig. 1), the year-round surface warming rate ranges from 0.40 and 0.49 °C/decade for estimates from simple linear regression, the Theil–Sen estimator, and the STL (Table 1, Figs. 2a and 3a, b). These values suggest that the mooring location is not thermally distinct from the long-term trend of the lake. Although the different statistical approaches were consistent regarding the long-term trend, the STL reveals an interannual variability that coincides with recognized warm and cool years (Fig. 3b)^31,32^. Broken down into monthly trends, linear analysis shows the greatest surface warming rate occurs in October, though warming trends are found from January through April as well (Fig. 4). Monthly trends from the STL confirm a surface warming trend in September and October with upticks occurring after 2010, though winter trends at the surface are relatively flat (Fig. 5a).

Subsurface observations detail the translation of surface warming trends through the water column. The time series of subsurface temperature resembles a heartbeat pattern (Fig. 1b); the annual rise and peak in temperature coincides with the fall overturn, where the warm water from above is mixed down and arrives at depth. The subsequent temperature dips reveal the annual winter cooling periods, and thus unlike the surface, subsurface temperatures only fluctuate between fall overturn and the onset of summer stratification. Long-term warming trends exist below the thermocline at the 60, 75, 100, and 110 m transects (Table 1). At the 60–100 m depths, the warming trends are less than the surface but relatively consistent across depths and among statistical approaches. At the 110 m transect, which includes the cool period in 2014 and 2015, trends are roughly half of the shallower subsurface trends (Table 1). Linear estimates suggest warming rates of 0.05 and 0.04 °C/decade for simple linear regression and Theil–Sen methods, respectively (Table 1, Fig. 3e). 95% confidence bounds for both estimates include zero slope, suggesting a possible nonlinear long-term trend at this depth. The STL analysis arrives at a slightly higher overall warming rate, 0.06 °C/decade, but clearly shows a high degree of interannual variability and reinforces the nonlinearity of the temperature trends at 110 m (Table 1, Fig. 3f). Below this depth, at the 140 m transect, no trends are found among the linear and STL methods.

Similar to surface trends, subsurface temperatures reveal strong seasonal trends. As the summer stratified period is extended, indicated by fall warming trends at the surface, the fall turnover date is delayed, meaning the receipt of warmer waters at depth is delayed. This is noted by relative cooling trends below the thermocline in September at 30 m, and consequently in November for 60 and 75 m depths (Figs. 4, 5b–d). Below 60 m, peak warming occurs in the winter from January through April (Figs. 4, 5c–f). During this period, the water column has mixed and deep subsurface temperatures respond to winter atmospheric conditions. At 75 and 100 m, linear estimates suggest warming trends from January through August, however at 110 m, this is reduced to January and October (Fig. 4). Using a nonlinear approach, the STL confirms the relative cooling trends (extended stratification) in the fall at 30, 60, and 75 m (Fig. 5b–d), and winter warming trends at 30, 60, 75, 100, and 110 m (Fig. 5b–f). However, where linear estimates find no trend, the STL provides a more detailed picture by revealing which trends were essentially flat and which were highly nonlinear. For instance, at the 110 m depth, the period from February through April reveals significant short-term trends that occurred in the late 1990s (Fig. 5e).

**Fig. 6:**
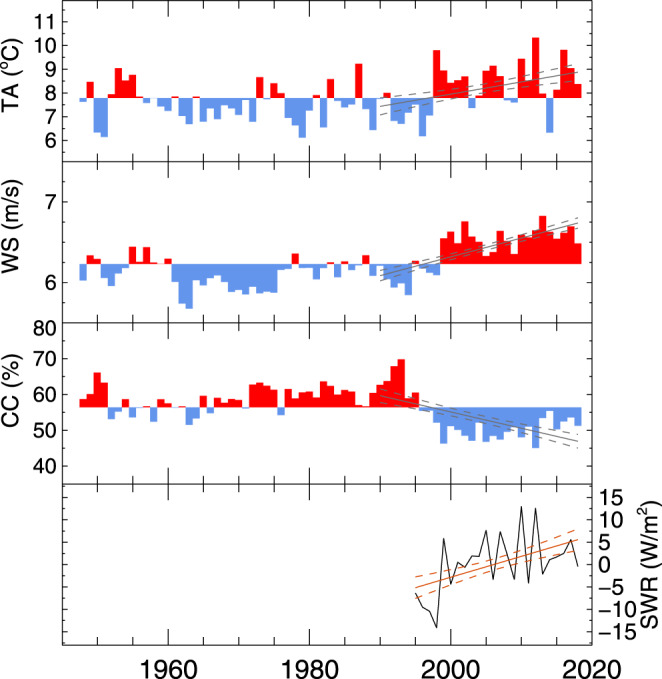
Time series of observed atmospheric conditions. Annual averages of 2 m air temperature (TA), 10 m wind speed (WS), cloud cover (CC), and downward shortwave radiation anomaly (SWR) show a change in the mid to late 1990s to a period of increased air temperatures and wind speeds, and decreased cloud cover (increase in shortwave radiation). For TA, WS, and CC, colors represent values that are above (red) or below (blue) their long-term annual mean. For the shorter time series record for SWR anomaly, annual averages are plotted directly. Linear regressions over the period of water temperature observations (1990–2018, except for SWR, which starts in 1995) estimate trends in TA (0.52 ± 0.22 °C/decade), WS (0.23 ± 0.04 m s^−1^/decade), CC (−4.55 ± 1.17%/decade), and SWR (4.66 ± 1.79 W m^−2^/decade).

**Fig. 7:**
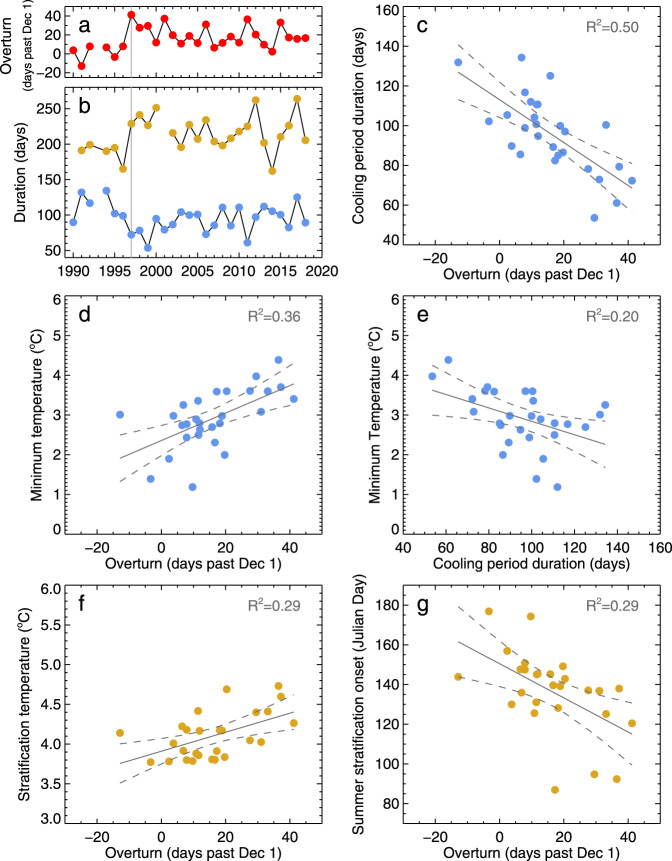
Relationships between overturn and deep water dynamics. **a** Overturn date at the 110 m transect, **b** cooling period duration (blue), and duration of summer stratification (gold) reveal shifts after 1997 (vertical gray line). **c** As the overturn date occurs later in the year (shown on *x*-axis), the cooling period duration decreases proportionally. With later overturn dates and shorter cooling periods, **d**, **e** the minimum temperature increases. Similarly, **f** the summer stratification temperature (the temperature at the inflection point defined as “S” in Fig. 1d) increases and **g** the date of summer stratification occurs earlier with delays in fall overturn.

The long-term warming trends in water temperature are consistent with observed changes in atmospheric conditions that occurred in the mid-1990s compared to their long-term means (Fig. 6). Over the duration of the water temperature record, increasing trends are found in overlake air temperature (0.52 ± 0.22 °C/decade), wind speed (0.23 ± 0.04 m s^−1^/decade), and shortwave radiation (4.66 ± 1.79 W m^−2^/decade), which is concurrent with a decrease in cloud cover (−4.55 ± 1.17%/decade). These trends are also consistent with increases in air temperature and the duration of summer stratification that have been reported in other lakes^7,9,10,33–35^, and a shift in several climate indices associated with the 1997–1998 El Niño^32,35^. Long-term means before (1948–1997) and after (1998–2018) the El Niño changed from 7.45 to 8.55 °C for air temperature, 6.10–6.54 m s^−1^ for wind speed, and 59.1–50.1% for cloud cover over the lake. As surface water temperatures respond directly to the increases in air temperature and solar radiation, these changes explain the extended summer-stratified period (warming trends in the fall) and the milder winter months indicated in Fig. 4. However, as deep water temperatures undergo warming and cooling phases between fall overturn and the onset of summer stratification, subsurface conditions are dependent on the intensity of winter atmospheric conditions and the duration of this period. Therefore, increased air temperatures and more incoming shortwave radiation (Fig. 6) that lead to less intense and shorter winters (Fig. 7a, b) will result in less overall subsurface cooling and consequently lead to the peak warming trends found from January through the spring (Fig. 4).

### Deep water cascade

In dimictic lakes, which collectively represent more than half of the world’s surface freshwater^1^, the water column undergoes two surface to bottom mixing events each year, one in the fall and one in the spring. As a dimictic lake, in Lake Michigan the surface cools in the fall (Fig. 2c), the overturn causes a sharp rise in subsurface water temperatures (“O”, Fig. 2d), establishing a mixed water column. From this point, the bottom waters enter a cooling phase over the winter, as the water column becomes inversely stratified, until a point of minimum temperature is reached (“M”, Fig. 2d). As spring conditions bring increased surface heat flux into the lake, the water column becomes fully mixed again and bottom waters warm until summer stratification is reached (“S”, Fig. 2d). The time between the date of fall overturn and minimum temperature is the only cooling period experienced by deep waters.

In 30 years of subsurface observations in Lake Michigan, the overturn date at 110 m depth ranges from mid-November to early January (Fig. 7a). The duration of the annual cooling period and the stratification period can be calculated on the timing from O to M and S to O, respectively (Fig. 7b). Cooling period duration ranges from 53 to 134 days and summer stratification varies between 162 and 263 days. In all three time-series, a notable shift occurred in 1997–1998 (Fig. 7a, b). The overturn date slid from early to late December, while the subsequent cooling period shortened to below 100 days and summer stratification extended beyond 200 days. Consistent with shifts in overlake meteorological conditions (Fig. 6), this shift appears to extend throughout the remainder of the water temperature record, although there is high interannual variability, particularly in the duration of summer stratification in recent years. Corroborating these findings, recent studies have identified shifts in several physical indices in the Great Lakes region associated with the 1997–1998 El Niño, including changes in incident radiation, ice cover, and teleconnections patterns such as the Pacific Decadal Oscillation (PDO)^31,32^. Although this subsurface record only extends back to 1990, a modeling investigation of Lake Michigan heat content that extended back to 1950 supports the notion that the lake underwent a jump in heat content in the late 1990s^31^ tied to the noted changes in atmospheric conditions (Fig. 6). A similar jump in surface temperature and increase in the duration of the summer-stratified period has also been reported in Lake Superior^35^.

The delay in fall overturn is the first step in a cascade of subsurface dynamics that signifies the impacts of surface warming on bottom waters. As the overturn date is delayed, we find a shortening in the duration of the subsequent cooling period (Fig. 7c). The shortened winter tends to result in higher minimum bottom temperatures, with the exception of the four coldest winters (Fig. 7d, e). These minimum winter temperatures, which fall near or below 2 °C, occurred in 1996, 2003, 2014, and 2015 (Fig. 2b). Not surprisingly, such conditions suggest that in addition to cooling period duration and turnover date, meteorological forcing during the winter plays a critical role on the minimum temperature at depth. Overall, the period of record reveals a remarkable range of minimum temperatures from 1.2 to 4.4 °C at 110 m (Fig. 7d, e). As conditions warm in the spring, the water column again becomes mixed and then summer stratification is established. In the final stage of this cascade, increases in bottom stratification temperature, above the temperature of maximum density, and the onset of summer stratification correlate with delays in the previous fall turnover (Fig. 7f, g). This signals the impact of winter conditions on the subsequent summer. However, with increasing time from the fall overturn date, these relationships show weaker correlations (Fig. 7c, d, f, g), wherein the cumulative impacts of the meteorological conditions during this period, or short-term natural weather variability, would become increasingly important. In all, these observations detail the connectivity between different deep water dynamics and the expected response to surface warming.

### Whither leads the warming in lakes?

The primary physical response of lakes to climate change will manifest as changes in water temperature, ice cover, water storage, and thermal structure^4,36^. Changes in subsurface temperatures can result in changes to water column stability, mixing, and duration of stratification^2,34,37,38^. However, long-term and high-resolution measurements are difficult to acquire, particularly for year-round sampling in Earth’s largest and deepest lakes. The unique long-term data set presented here extends the conclusions of previous studies^13,18^ by investigating subsurface temperatures and trends across seasons, confirming that surface warming is translated to deep waters in a large lake. Using hourly measurements, the impacts of delays in fall overturn and extension of stratification become clear. In response to these timing changes, more so than just surface warming itself^39^, the subsurface conditions are altered in the winter and spring. These dynamics illustrate the pathway for mixing regime shift in large lakes^2^.

Projections of climate-driven impacts on lakes show the likelihood of mixing regime shifts by the end of this century^15^. Models reveal the potential decrease in ice cover and increase in surface water temperatures. In large dimictic lakes, the delay in fall overturn, loss of ice, and collapse of the cooling period can push the regime to become warm monomictic. Not all lakes that experience warming temperatures are projected to undergo a mixing regime shift, as meteorological and other conditions can mitigate the effects, but for those that do, many exhibit similar traits^15,40^. For dimictic lakes, this includes those that currently have seasonal ice cover but experience occasional ice-free winters. Like many of Earth’s large lakes, Lake Michigan meets the criteria for a dimictic to warm monomictic shift, with a decreasing long-term trend in ice cover and several low-ice or nearly ice-free years since 1990^41,42^. Here we present the deep water relationships that may lead to this transition in response to climate change.

The implications of mixing regime shifts in large lakes are well documented. The extension of summer stratification can reduce dissolved oxygen replenishment to deep waters and exacerbate hypoxic conditions^43^. Reorganization of food web structure and shifts in dominant species, including the possible proliferation of non-native invasive species, are possible effects of thermal change^29,44^. Already in Lake Tanganyika, increases in water temperature are responsible for reductions in primary productivity^12,14,23^. Conversely, warming temperatures and decreasing ice cover in Lake Superior are driving an increase in primary productivity^45^. In Lake Baikal, the world’s deepest large lake, the changing thermal conditions are resulting in community shifts among zooplankton^10^. In all, the consequences of changes in subsurface water temperatures will result in a profound shift in lake ecology. Without high-frequency long-term monitoring of subsurface waters, we will be blind to the impacts of climate change on most of Earth’s fresh surface water.

## Methods

### Water temperature data

Detailed vertically distributed water temperature data are used to analyze long-term trends and deep water dynamics in Lake Michigan^46^. Subsurface observations began in 1990 at a 150 m deep location in the central southern basin of the lake (42°40.493′N, 87°04.772′W; Fig. 1). The site was chosen due to depth, proximity to the National Oceanic and Atmospheric Administration (NOAA) National Data Buoy Center (NDBC) buoy 45007, and vessel access. The deployment date, number of sensors, and sensor depths vary by year (Supplementary Information). From 1990 to 1994, Aanderaa TR7 thermistor chains were deployed with 3-hourly measurements, which have an accuracy of ±0.03 °C. For these years, the depths of each sensor were calculated from total water column depth and the measured distances from the bottom of the chain. Sensor malfunction between 1993 and 1994 led to a large data gap. From 1994 to 2012, individual sensors were deployed in place of the TR7 chains to capture water temperatures at hourly intervals (TR-1000 by Richard Brancker Research Ltd. (RBR) with accuracy of ±0.05 °C). For this period, a pressure sensor was added to the uppermost sensor and depths were calculated based on measured distance from this sensor. From 2012 to 2019, the sensors were changed to Sea-Bird Scientific SBE56 (accuracy ±0.002 °C). In 2013, a faulty mid-line subsurface float led to the sinking of the thermistor string. However, the upper subsurface float allowed the top nine sensors to be suspended over the bottom 40 m of the water column. Each year the data were downloaded from the sensors using software provided by the manufacturer and the data were manually corrected to remove inaccurate values. When necessary due to primary sensor malfunction, the Tidbit sensor data was used to fill data gaps.

Temperatures at the water surface were acquired from the Great Lakes environmental surface analysis (GLSEA)^30^ for the period 1995–2018 (*n* = 8743), a data set commonly used for long-term trends in Great Lakes surface temperatures^16,17^. The GLSEA is a 1.8 km resolution satellite-derived surface temperature product that provides daily lake surface temperatures throughout the year. The data set is based on the NOAA advanced very high-resolution radiometer (AVHRR), a sensor aboard NOAA’s Polar Orbiting Environmental Satellites (POES).

### Atmospheric data

Meteorological observations of air temperature, cloud cover, and wind speed are used to examine the atmospheric drivers of water temperature trends in Lake Michigan. Overlake conditions are acquired from the Great Lakes Coastal Forecast System (GLCFS^47^), which has hourly observations back to 1988. To put recent changes in context with long-term trends, these data are supplemented with the Great Lakes Monthly Hydrometeorological Database (GLM-HMD^48^) for the period 1948–1987, which provides daily overlake conditions for each of the Great Lakes. Both data sets use several over-water and coastal meteorological stations that include NOAA Coastal-Marine Automated Network (C-MAN) sites, National Weather Service buoys, the Automated Surface Observing System (ASOS), and United States Coast Guard Stations^49^. In this study, we use sites in close proximity to Lake Michigan (Fig. 1), in which the GLCFS applies atmospheric adjustments to a common measurement height and employs a natural neighbor interpolation^50^ to create hourly overwater meteorological conditions on a 2-km grid, which are then spatially averaged to provide lake-averaged conditions for each variable. GLCFS data are averaged into daily conditions and then both datasets (GLCFS and GLM-HMD) are averaged into annual values. For comparison to overlake cloud cover conditions, downward shortwave radiation is acquired from the NOAA Surface Radiation (SURFRAD) Network station located at Bondville, Illinois (Fig. 1)^51^. Monthly averages of shortwave radiation from the SURFRAD station are averaged into annual values for the period 1995–2018. Long-term means are calculated for air temperature, cloud cover, and wind speed, whereby annual averages are plotted in comparison to their long-term means. Since the shortwave radiation data only extend back to 1995, a long-term mean was not calculated. For each parameter, a linear regression and confidence intervals (one standard deviation) are calculated for the period of the water temperature record (1990–2018, except shortwave radiation which starts in 1995) to highlight the atmospheric mechanisms behind the reported water temperature trends.

### Data analysis and seasonal trend decomposition

For data analysis purposes, sensor depths were defined as constants for each year based on the mean depth of the deployment period. Time series data was extracted and interpolated to specific depths (30, 60, 75, 100, 110, 140 m with resulting sample sizes of 207,958, 204,914, 208,904, 207,619, 222,251, and 186,731, respectively), chosen to cover the full range of the water column and for proximity to sensor locations. For these transects, temperature data was linearly interpolated from the nearest upper and lower sensors. In the case of the 110 m transect, an effort was made to include the early years from 1990 to 1993 as well as the period 2013–2015 to produce the longest possible data record at depth. For this depth, the bottom sensor in 1990–1993, located at 108 m, and from the uppermost sensor in 2013–2015, located at 111 m, were used to complete the record, where all other periods were interpolated as noted above. To check for consistency, we conducted a sensitivity analysis to ensure the trends and relationships results presented are not unique only to the chosen transect depths. This analysis consisted of using additional interpolated depths to assess subsurface long-term and monthly trends (at 25, 50, 120, and 125 m depths) for the results presented in Table 1 and Figs. 3–5, and Supplementary Information. Similarly, for the deep-water relationships presented in Fig. 7, data from the 100 m transect was employed to verify the shift noted after 1997 (Fig. 7a, b) and the strength of the relationships (Fig. 7c–g). In all cases, the choice of transect depth did not significantly deviate from the long-term and monthly trends presented, nor did it impact the relationships presented in Fig. 7.

Analyses of long-term and monthly trends were performed using three statistical approaches, simple linear regression, Theil–Sen estimators, and seasonal trend decomposition using loess (STL)^52^. Linear regressions and Theil–Sen estimators are common approaches for trend analysis, but as the inherent trends are not linear, we have supplemented the analysis with the STL decomposition. The combination of methods can confirm existing trends, unveil nonlinearities or where trends are nonexistent, and differentiate long-term trends from seasonal and residual components. Confidence intervals are provided using parametric and non-parametric (bootstrapping by sampling with replacement) methods for the linear regressions and Theil–Sen estimates, while linear regressions of the STL long-term trend was employed to provide an overall trend and confidence interval. In preparation for monthly analysis, the data were averaged for each day and then over each month to obtain monthly averaged water temperatures at the surface and chosen subsurface transects.

The STL method has been used for long-term trend analysis in large lake tributary inflows and nutrient concentrations^53,54^. With the STL approach, monthly averaged water temperatures were decomposed into long-term, seasonal, and residual components (Figs. 3 and 5). A single loess line was fitted to the long-term component and similarly, 12 monthly loess lines were used in the seasonal component. Iterations are performed until convergence is reached, meaning the long-term and seasonal components no longer differ from the previous iteration. Window widths are chosen to produce smoothed long-term patterns, reduce edge effects, and reduce the residuals component.

For linear trend analysis of monthly data at depth (Supplementary Information), slopes from simple linear regressions of monthly data are used to illustrate monthly warming and cooling trends in the water column (Fig. 3). Each month’s trend is normalized by the maximum absolute value of the monthly trends for a given depth, such that the darkest red for a particular depth indicates the month of greatest warming for that transect during the period 1990–2019. For Fig. 3, confidence intervals on the linear trends are calculated at 1.96, 1.645, and 1.0 standard deviations and indicated by gold, half-gold, and white circles, respectively.

For analysis of deep water dynamics, we chose the water temperature record at the 110 m transect in an attempt to include longest possible record over the 30-year period, though the results presented were verified at the 100 m as noted in the sensitivity analysis described above. In each year, when possible, the overturn date was defined as the point of maximum temperature on the spike in water temperature at 110 m (Fig. 2d). Similarly, the minimum temperature for each year was found for the period following the overturn date. The cooling period is defined as the number of days from the overturn date to the date of minimum temperature. The stratification temperature and date is found at the inflection point after the point of minimum temperature, where the water temperature ceases to warm and becomes relatively constant over the stratified season. The duration of the stratified season is defined as the number of days from the stratification date to the following fall overturn date. For clarity, the overturn dates were referenced relative to December 1. Linear least-square fits were shown as a visual guide for the observed relationships between deep water variables along with a calculated square of the Pearson correlation coefficient, *R*^2^, and parametric 95% confidence intervals (Fig. 7).

## Supplementary information

Supplementary Information

## Data Availability

The subsurface water temperature data used in the study are available in the NOAA National Centers for Environmental Information Archives with the accession codes 0190726 and 0203568. Satellite-derived surface water temperature data is available from the NOAA Coastwatch (https://coastwatch.glerl.noaa.gov/thredds/Satellite/glsea/glsea_catalog.html). Meteorological observations are available from the NOAA, CoastWatch Great Lakes Node (NOAAPORT Realtime Great Lakes Weather Data and Marine Observations. Retrieved from https://coastwatch.glerl.noaa.gov/marobs/. Accessed 10/23/2020). Shortwave radiation observations are available from the NOAA Earth Systems Research Laboratory Global Radiation Surface Radiation Budget (SURFRAD) Quality Control Radiation (QCRAD) Level 3 Measurements, Version 1. (ftp://aftp.cmdl.noaa.gov/data/radiation/surfrad/averages/) Accessed: 10/23/2020.

## Code availability

Data analysis was performed using R statistical language and figures were created using IDL programming language (Harris Geospatial). Code used for STL analysis is available at https://github.com/songsqian/GL_STL.
