# Supplementary material for: Seasonal overturn and stratification changes drive deep-water warming in one of Earth’s largest lakes
Source: Nat Commun. 2021 Mar 16;12:1688. doi: 10.1038/s41467-021-21971-1 (PMC7966760; doi:10.1038/s41467-021-21971-1)
Supplement: Supplementary file 1 — Supplementary Information [file 41467_2021_21971_MOESM1_ESM.pdf]

Supplementary Information for

Seasonal overturn and stratification changes drive deep-water warming in one of Earth's largest lakes

Eric J. Anderson[1*], Craig A. Stow[1], Andrew D. Gronewold[2], Lacey A. Mason[1], Michael J. McCormick[1], Song S. Qian[3], Steven A. Ruberg[1], Kyle Beadle[1], Stephen A. Constant[1], Nathan Hawley[1]

[1*]Great Lakes Environmental Research Laboratory, Office of Oceanic and Atmospheric Research, National Oceanic and Atmospheric Administration
[2]School for Environment and Sustainability, University of Michigan
[3]Department of Environmental Sciences, The University of Toledo

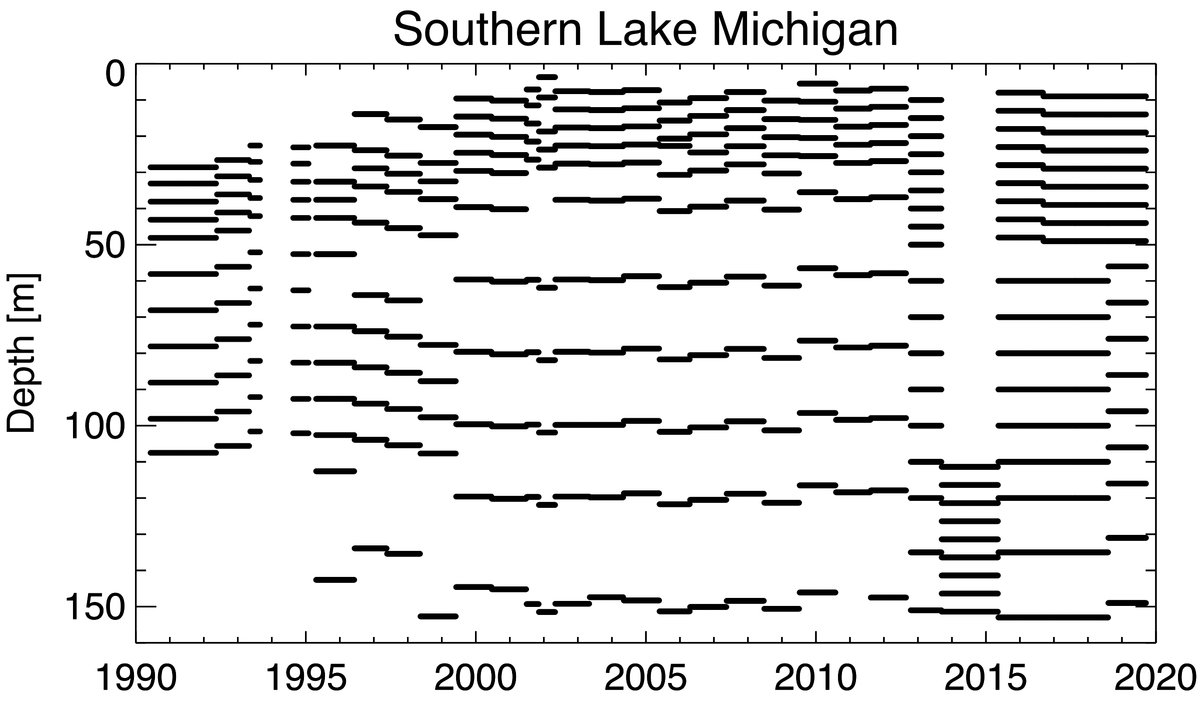

Supplementary Figure 1: Lake Michigan long-term subsurface temperature mooring. Thermistor sensor depths for each deployment indicated by black dots/lines for the thirty-year period.

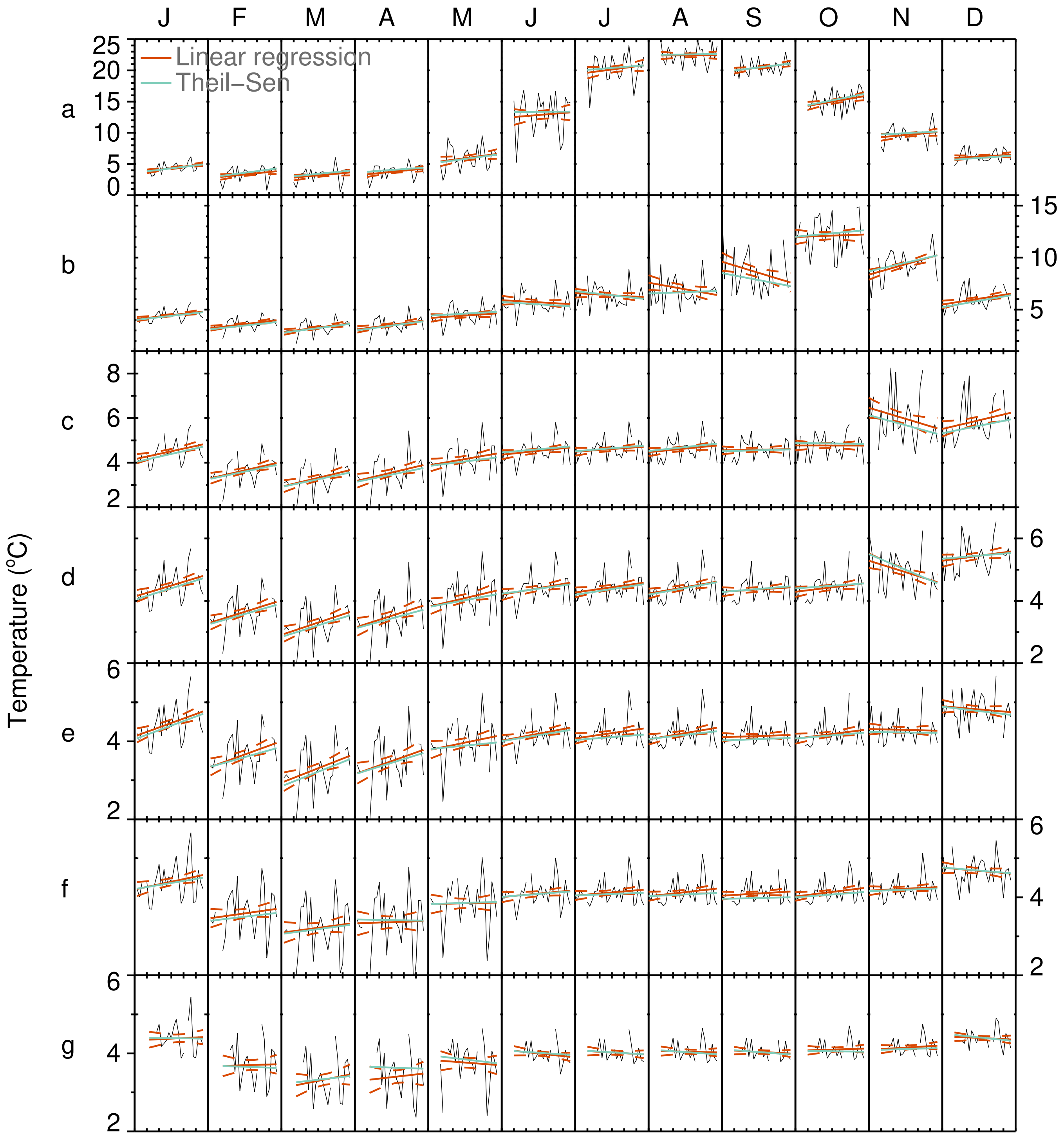

Supplementary Figure 2: Linear trends of monthly water temperatures. Time-series of water temperature data separated into months, and linear trends computed by simple linear regression and Theil-Sen estimators, for the **a**, surface, **b**, 30 m, **c**, 60 m, **d**, 75 m, **e**, 100 m, **f**, 110 m, and **g**, 140 m transects. Slopes from the simple linear regressions are used to indicate warming and cooling trends in Figure 3. For each month, the x-axis represents the range 1990 – 2019.
